# The prognostic influence of hospital type, method of first histological confirmation and time to chemotherapy in patients with advanced primary ovarian cancer

**DOI:** 10.1007/s00404-024-07832-4

**Published:** 2024-12-16

**Authors:** Olivia Starke, Pauline Wimberger, Daniel Martin Klotz

**Affiliations:** 1 Department of Gynecology and Obstetrics, Technische Universität Dresden, Fetscherstraße 74, 01307 Dresden, Germany; 2 National Center for Tumor Diseases/University Cancer Center (NCT/UCC): German Cancer Research Center (DKFZ), Heidelberg, Germany, Faculty of Medicine and University Hospital Carl Gustav Carus, Technische Universität Dresden, Helmholtz-Zentrum Dresden-Rossendorf (HZDR), Dresden, Germany; 3 German Cancer Research Center (DKFZ), Heidelberg and German Cancer Consortium (DKTK) Partner Site, Dresden, Germany

**Keywords:** Advanced ovarian cancer treatment, Port site metastases, Centralization, Time to chemotherapy, Tertiary hospital, Non-tertiary hospital

## Abstract

**Purpose:**

Ovarian cancer is the fifth most common cancer in women and the leading cause of death of all gynecological malignancies. Prognosis is determined by optimal surgical outcome (macroscopic complete resection) most commonly achieved in tertiary hospitals. We investigated whether tertiary versus non-tertiary hospital as the location of an initial diagnostic intervention for histological confirmation before cytoreductive surgery versus immediate primary debulking surgery impacts outcome in patients with advanced ovarian cancer.

**Methods:**

We analyzed 115 patients who underwent cytoreductive surgery at a German tertiary center: 60 patients underwent primary debulking surgery (PDS) and 55 patients had a diagnostic intervention for histological confirmation before debulking surgery (PHC).

**Results:**

Although there was no prognostic difference between the two subgroups, the median time to chemotherapy was longer in the PHC group (46 days) compared to the PDS group (26 days; *p* < 0.0001), equally seen comparing non-tertiary versus tertiary PHC groups (*p*: 0.0001), its impact confirmed in a multivariate analysis (PFS: HR: 1.03, 95%CI: 1.01–1.05, *p*: 0.007; OS: HR: 1.04, 95%CI: 1.02 –1.06, *p*: < 0.001) of the PHC group only. In total, 9/10 patients with port-site metastases after diagnostic laparoscopy were initially treated at non-tertiary hospitals, resulting in a lower PFS compared to patients without port-site metastases after laparoscopy (HR 0.21, 95%CI 0.06–0.733, *p*: 0.014).

**Conclusions:**

In conclusion, patients with ovarian cancer undergoing treatment solely at a tertiary center have some clinical benefits and improved outcome, given the shorter time to chemotherapy and potential impact of port-site metastases. This supports centralization of oncological treatment.

**Supplementary Information:**

The online version contains supplementary material available at 10.1007/s00404-024-07832-4.

## What does this study add to the clinical work

**Table:** 

Our study shows that patients with advanced ovarian cancer undergoing treatment solely at a tertiary center have some clinical benefits, i.e. a shorter time to chemotherapy and a reduced rate of port-site metastases. Our study contributes to the ongoing debate about promoting centralization of oncologic treatment in specialized, certified centers.	

## Introduction

Ovarian cancer is the fifth most common women’s cancer, accounts for about 30% of malignancies of the female reproductive system and is the leading cause of death of gynecological malignancies in Germany [1].

The treatment typically includes primary surgical debulking with the aim to achieve a macroscopic complete resection followed by platinum- and paclitaxel-based chemotherapy (CTx). CTx is combined with a treatment targeting angiogenesis with bevacizumab for patients with FIGO III/IV, which is continued as maintenance therapy for 15 months in total. In patients with homologous recombination deficiency (HRD) or somatic/germline *BRCA1/2* (*Breast Cancer 1/ Breast Cancer 2*) mutations, maintenance treatment after response to platinum-based chemotherapy consists of Poly-ADP-Ribose-Polymerase-inhibitors (PARPi) olaparib in combination with bevacizumab [2]. Alternatively, patients can be treated with niraparib maintenance therapy after response to platinum-based CTx irrespective of biomarker status (HRD or *mBRCA1/2*) [3].

Patients diagnosed with early stages of ovarian cancer (FIGO I/II) have an excellent prognosis, but most patients are diagnosed at advanced stages partially due to a lack of appropriate screening methods. Throughout all stages there is a 5-year survival rate of about 42% [4]. However, it is important to note that new treatment regimens with maintenance treatment consisting of PARP inhibition and bevacizumab in patients with HRD or m*BRCA1/2* mutation after optimal surgical debulking have reported progression-free survival (PFS) at 2 years in more than 90% of patients [5]. These treatment have also been shown to improve overall survival (OS) in patients in this lower risk group (FIGO III, primary cytoreductive surgery with no residual postoperative tumor) [6]

Therapeutic treatment options have vastly improved in the last decade, yet the most important prognostic factor remains surgical outcome. In case of an immediate primary debulking surgery (PDS), this routinely involves the intraoperative histological confirmation of the disease. However, in some cases histological confirmation is obtained prior to cytoreductive surgery. Reasons may include (1) an incidental diagnosis during routine gynecological surgeries [7], (2) symptomatic ascites that requires ascites drainage, (3) possibility of non-ovarian malignancies, requiring different treatment regimens (i.e. patients with a previous history of breast cancer), (4) the assessment of the probability of complete surgical debulking in cases where it is uncertain that complete resection during primary debulking surgery is feasible [8].

These early diagnostic interventions may be performed in different hospital types. These procedures may commonly be performed at non-specialized hospitals and patients referred to a tertiary center once the diagnosis of ovarian cancer has been confirmed. Alternatively, a patient is primarily referred to a tertiary center and all diagnostic, surgical interventions and treatments are performed there.

It could be possible that different types of hospitals (non-tertiary *versus* tertiary centers) at the time of primary diagnosis may lead to differences in clinical outcome. This could result in worse prognosis, increased morbidity, or delay time to subsequent therapies of individual patients.

Therefore, we wanted to test the hypothesis that the place where patients receive the initial diagnostic intervention for histological confirmation of ovarian cancer prior to primary debulking surgery results in clinically relevant prognostic differences. We analyzed a panel of clinical parameters, including the type of surgical/diagnostic intervention prior to primary surgical debulking, time to subsequent CTx, PFS, OS and the occurrence of port-site-metastases.

## Methods

In this retrospective study, we analyzed all patients with ovarian cancer that underwent cytoreductive surgery with the aim of macroscopic complete resection at the Department of Gynecology and Obstetrics, University hospital Dresden (UKD), TU Dresden, Germany between 2015 and 2018. The analysis was approved by the local Ethics committee (EK-354082022).

Inclusion criteria were histologically confirmed primary ovarian cancer (or primary fallopian tube cancer or primary peritoneal cancer), with cytoreductive surgery with the aim of a macroscopic complete resection performed at the UKD followed by adjuvant CTx. Participation in clinical trials was allowed. Exclusion criteria were Fédération Internationale de la Gynécologie et de l’Obstétrique (FIGO) I/II stages, neoadjuvant CTx and borderline tumors.

### Statistical analysis

The statistical analysis was conducted with GraphPad prism version 8.4.3 (GraphPad Software, La Jolla, CA, USA) and with “R Version 4.4.1 *p* values < 0.05 were considered statistically significant. All confidence intervals (CIs) were specified as 95% CI. Non-parametric, two-tailed Mann–Whitney *U* test was used to compare different patient subgroups. Progression free survival was analyzed as recurrence or death with the primary histological confirmation as starting point and shown with interquartile range. Overall survival was analyzed as death or last follow up with the primary histological confirmation as starting point. The R package “MatchIt” was used to perform case–control matching. The Kaplan–Meier analyses were performed with significance levels indicated by log-rank (Mantel–Cox) analysis, and HRs (Mantel–Haenszel) are shown with 95% CI. Uni‐ and multivariate Cox proportional hazard regression model analyses were performed to study the prognostic relevance. Hazard ratios (HRs) are indicated with 95% CI.

## Results

### Patients’ cohort

In total, 169 patients received primary cytoreductive surgery in the specified time period of which 115 were included based on the above-mentioned criteria. Our cohort was split in two groups: The first group (n: 60) received primary debulking surgery (PDS group) without prior histological confirmation at the time of surgery, but with radiological, sonographic and tumor marker evaluation, typically assessed by the IOTA criteria [9]. The other group included patients (n: 55) who received a diagnostic/surgical intervention within 6 months prior to the cytoreductive surgery, referred to as previous histological confirmation (PHC) and PHC group. These interventions included: 29 laparoscopies (LSC), 7 laparotomies, 6 CT-guided biopsies, 11 ascites taps, and two others (1 peritoneal biopsy and 1 lymph node resection).

Key clinical characteristics were equally matched in the PDS and PHC groups. There was no statistically significant difference in the median age, BMI, FIGO stages, the histological subtypes, the rates of macroscopic complete resection, nor in the CA125 prior to cytoreductive surgery (*p* > 0.05, Table 1). The end of follow-up was the 30th of March 2024. The median follow-up time was 70 months.

**Table 1:** Patient characteristics

Patient subgroups	Primary debulking surgery	Previous histological confirmation
n	60	55 (non-tertiary center *n* = 33; tertiary center *n* = 22)
Age	Median 62 years (54–73 years)	Median 63 years (53–71 years)
BMI	Median 25.1 (22.2–29.2)	Median 25.5 (23–29.4)
FIGO		
III	40 (66.7%)	33 (60.0%)
IV	20 (33.3%)	22 (40.0%)
Histological subtype		
High grade serous	53 (88.3%)	42 (76.4%)
Others	7 (11.7%)	13 (23.6%)
Progressionfree survival		
PFS	Median 19 months (10–48 months)	Median 24 months (9–49 months)
No recurrence	20 (33.3%)	21 (38.2%)
Recurrence/death	40 (66.7%)	34 (61.8%)
Overall survival		
OS	Median 44 months (18.75–63 months)	Median 54 months (12–63.5 months)
Alive	24 (40%)	28 (50.9%)
Deceased	36 (60%)	27 (49.1%)
Residual tumor		
Macroscopic complete resection	28 (46.7%)	23 (41.8%)
Post-operative tumor burden	32 (53.3%)	32 (58.2%)
CA125 [U/mL] before debulking surgery	Median 883.0 U/mL (341.8–3309 U/mL)	Median 660.3 U/mL (172.5−1991 U/mL)

For the subgroup analysis within the PHC subgroup, histological confirmation was performed at the tertiary center in 22/55 patients (40% of all PHC patients), whereas 33/55 patients (60% of all PHC patients) received the initial histological confirmation at non-tertiary medical centers. These subgroups are referred to as tertiary-PHC or non-tertiary-PHC and were matched 1:1 for the case–control analysis, i.e. *n* = 22 *versus* *n* = 22.

LSC was the most frequent surgical procedure prior to surgical debulking in the PHC group. We looked at this sub-cohort and the different type of hospital of the initial LSC, referred to as tertiary-PHC-LSC (i.e. patients solely treated at the tertiary center) and non-tertiary-PHC-LSC (i.e. patients treated first in a non-tertiary medical center and then referred to the tertiary center).

### Time to chemotherapy and time to definitive primary debulking surgery

There was no prognostic difference between the PDS group and the PHC group concerning the PFS (HR: 0.813, 95% CI: 0.505–1.308, *p*: 0.393) and the OS (HR: 0.713, 95% CI: 0.428–1.19, *p*: 0.194).

**Fig. 1:**
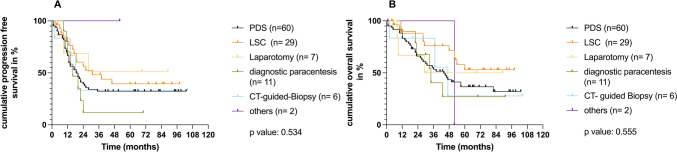
Prognostic relevance of different methods of histological confirmation. Kaplan–Meier analysis is shown for **A** Progression-free survival (PFS) of the primary debulking surgery group (PDS) in black compared to all the subgroups in the primary histological confirmation group (PHC) with number of patients in each group shown. **B** Overall survival (OS) of the primary debulking surgery group (PDS) in black compared to the primary histological confirmation group (PHC) here subdivided in its various subgroups with number of patients in each group shown. The *p* value is shown by log-rank (Mantel–Cox), as described in the methods section

Next, we assessed whether the type of diagnostic intervention prior to surgical debulking in the PHC group affects the outcome. The different procedures included 29 laparoscopies (LSC), 7 laparotomies, 6 CT-guided biopsies, 11 ascites taps (diagnostic paracentesis) and others (1 lymph node resection and 1 peritoneal biopsy). There was no statistically significant difference in the PFS (*p*: 0.534) or the OS (*p*: 0.555) between all the different subgroups of the PHC group and the PDS group (Fig. 1).

**Fig. 2:**
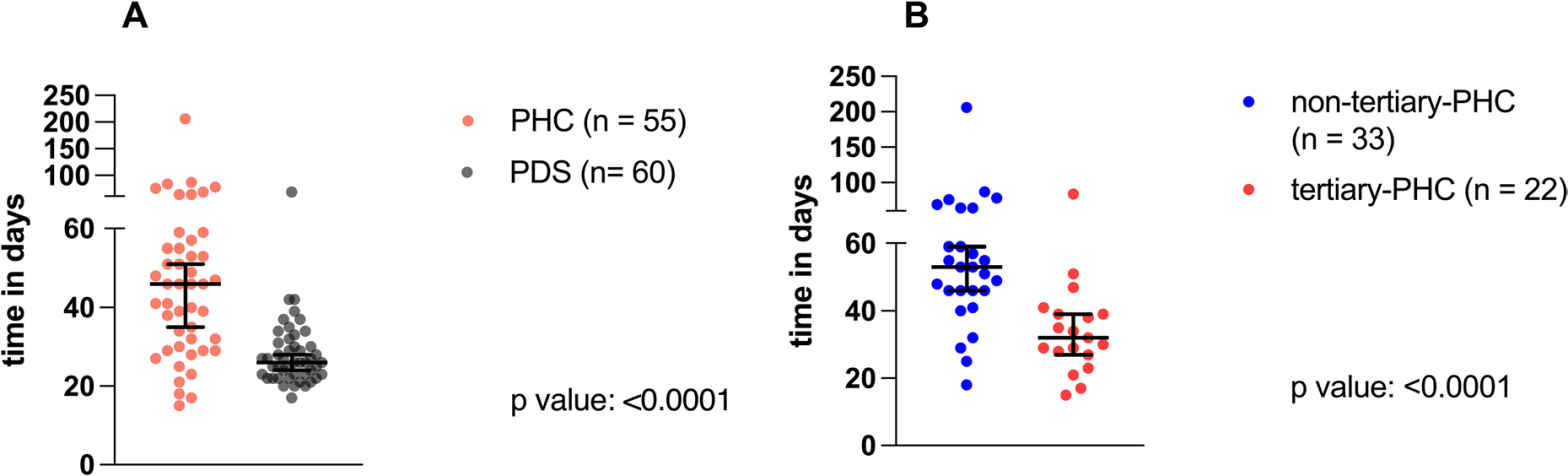
Time to chemotherapy from the day of the diagnosis to the first day of adjuvant chemotherapy. **A** Scatter plot comparing the time to chemotherapy (days) of the primary histological confirmation group (PHC; n: 55) and the primary debulking surgery group (PDS; n: 60). The black horizontal lines indicate the median time to chemotherapy in each group, with error bars showing the 95% CI. *P* value according to the non-parametric, two‐sided Mann–Whitney test. **B** Scatter plot comparing the time to chemotherapy (days) of the primary histological confirmation at a non-tertiary hospital (non-tertiary-PHC) and the primary histological confirmation at a tertiary hospital (tertiary-PHC). The black horizontal lines indicate median time to chemotherapy in days in each group, with error bars showing the 95% CI. *P* values according to the non-parametric, two‐sided Mann–Whitney test

However, time to CTx (calculated from the day of the histological confirmation to the start of adjuvant CTx) was significantly longer in the PHC group (median: 46 days) compared to the PDS group (median: 26 days) (Fig. 2 A), *p* < 0.0001).

### Time to chemotherapy at tertiary *vs.* non-tertiary centers and its prognostic significance in a multivariate analysis

There was no prognostic difference between the tertiary-PHC group and the non-tertiary PHC group (PFS: HR: 1.109, 95% CI: 0.534–2.306, *p*: 0.781; OS: HR: 0.976, 95% CI: 0.431–2.211, *p*: 0.954). This was also confirmed in the case–control matching analysis because there was no statistically significant difference between cases and controls in the PFS (HR: 0.788, 95% CI: 0.359–1.73, *p*: 0.547) or the OS (HR: 0.865, 95% CI: 0.368–2.04, *p*: 0.739). There were also no differences comparing either cases or controls to the PDS group (Supplementary Fig. 2 A-D).

Importantly, the time interval to adjuvant CTx differed between these subgroups with the non-tertiary-PHC group having a significant delay to CTx, measured as the day of the initial histological confirmed diagnosis to the first day of adjuvant CTx (non-tertiary-PHC: median time to CTx: 53 days *vs.* tertiary-PHC: median time to CTx: 32 days; *p*: 0.0001, Fig. 2 B).

Focusing on the PHC group and the clinical significance of solely tertiary *versus* initial non-tertiary care, we conducted a univariate and multivariate analysis of the following factors: non-tertiary *versus* tertiary PHC, FIGO stage, postoperative tumor burden and time to CTx (Table 2 Supplemental Tables 1 and 2). In the multivariate analysis we confirmed that the macroscopic complete resection had the most significant prognostic impact (PFS: HR: 12.02, 95% CI: 3.55–40.67, *p* < 0.001; OS: HR: 28.4, 95% CI: 4.99–161.69; *p* < 0.001). Although the hospital type of the primary histological confirmation (tertiary *vs.* non-tertiary) showed no prognostic difference, the time to CTx showed a prognostic difference in the multivariate analysis (PFS: HR: 1.03, 95% CI: 1.01–1.05, *p*: 0.007; OS: HR: 1.04, 95% CI: 1.02–1.06, *p* < 0.001) (Table 2).

**Table 2:** Multivariate Cox proportional hazard regression model analyses of the PHC group

Non-tertiary-PHC *vs*. tertiary-PHC	HR: 0.65; 95% CI: 0.27–1.56; *p*: 0.338	HR: 0.6; 95% CI: 0.23–1.57; *p*: 0.3
FIGO Stage IV *vs.* III	HR: 1.13; 95% CI: 0.5–2.57; * p*: 0.77	HR: 0.76; 95% CI: 0.3–1.93; *p*: 0.564
Postoperative residual disease *vs.* macroscopic complete resection	HR: 12.02; 95% CI: 3.55–40.67; *p*: < 0.001	HR: 28.4; 95% CI: 4.99–161.69; *p*: < 0.001
Time to chemotherapy	HR: 1.03; 95% CI: 1.01–1.05; *p*: 0.007	HR: 1.04; 95% CI: 1.02–1.06; *p*: < 0.001

The prognostic relevance for PFS and OS analyzed for: tertiary vs. non-tertiary primary histological confirmation, FIGO stage, postoperative tumor residuals and time to chemotherapy. Hazard ratio (HR) and 95% CI interval with p-values shown

### Laparoscopy and the occurrence/diagnosis of subsequent port-site metastases in different hospital types

There was no difference in PFS (HR: 0.732, 95% CI: 0.415–1.29, *p*: 0.281) or OS (HR: 0.564, 95% CI: 0.309–1.03, *p*: 0.063) comparing patients with LSC as their PHC with patients in the PDS group.

Comparing the time to surgical debulking between the non-tertiary-PHC-LSC subgroup and the tertiary-PHC-LSC group, we observed a difference in time to surgical debulking (non-tertiary-PHC-LSC: median time: 26 days; tertiary-PHC-LSC: median time: 10.5 days; *p*: 0.0025) with a significantly shorter time to surgical debulking in the tertiary-PHC-LSC subgroup.

**Fig. 3:**
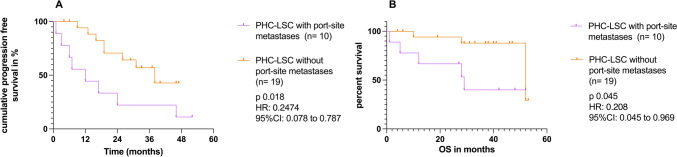
The prognostic relevance of port-site metastases. **A** Kaplan–Meier analysis showing progression-free survival (PFS) of the primary histological confirmation group who underwent laparoscopy and developed port-site metastases (PHC-LSC with port-site metastases; n: 10) compared to the primary histological confirmation group who underwent laparoscopy and did not develop a port-site metastases (PHC-LSC without port-site metastases; n: 19). **B** Kaplan–Meier analysis showing overall survival (OS) of the primary histological confirmation group who underwent laparoscopy and developed port-site metastases (PHC-LSC with port-site metastases; n: 10) compared to the primary histological confirmation group who underwent laparoscopy and did not develop port-site metastases (PHC-LSC without port-site metastases; n: 19). Kaplan–Meier curves with the HR and 95% CI determined by Mantel–Haenszel and *p* value by log-rank (Mantel–Cox), as described in the methods section

**Fig. 4:**
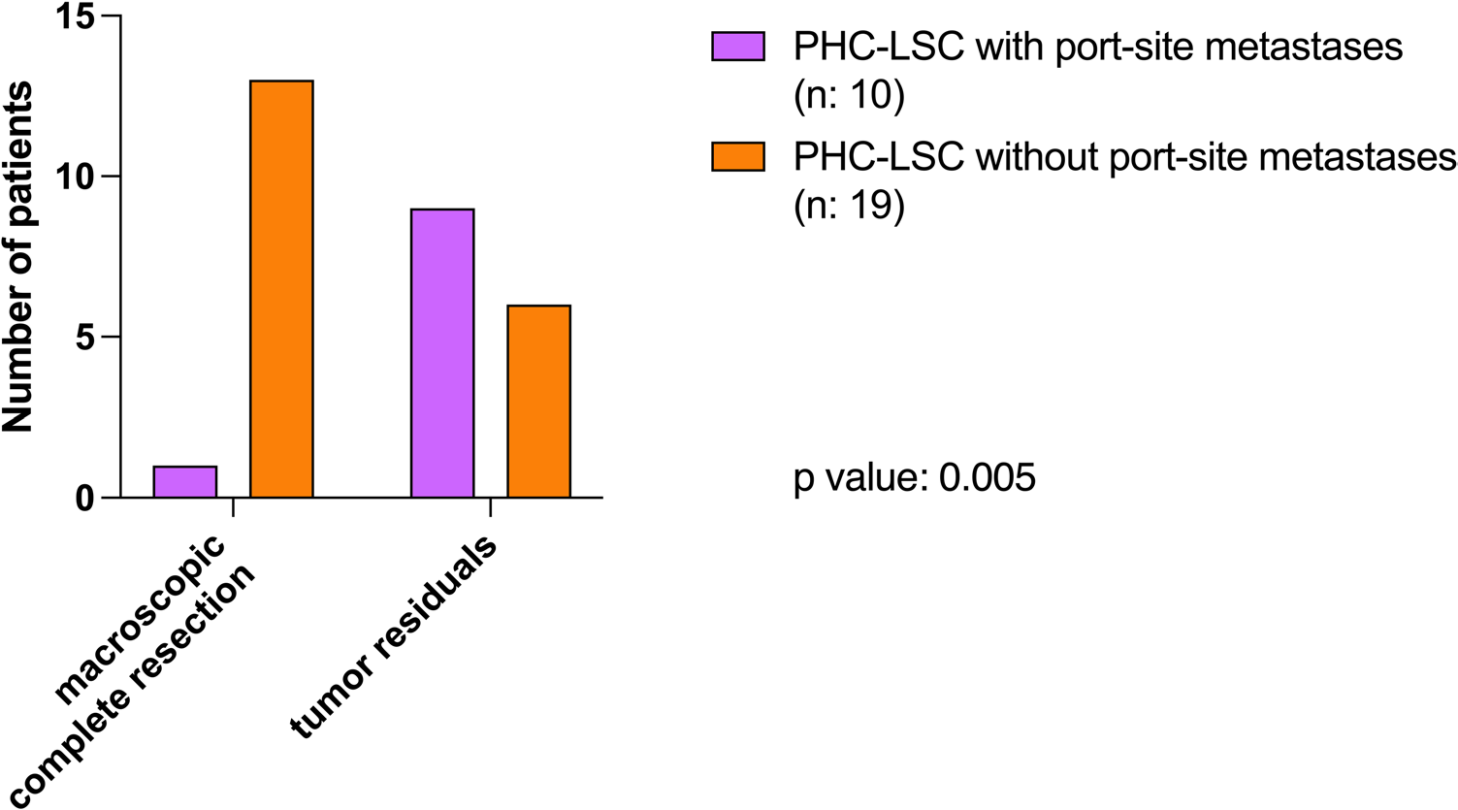
Macroscopic complete resection *vs.* tumor residual after debulking surgery. The graph shows the surgical outcome of the primary histological confirmation group who underwent laparoscopy and developed port-site metastases (PHC-LSC with port-site metastases; n: 10) compared to the primary histological confirmation group who underwent laparoscopy and did not develop a port-site metastases (PHC-LSC without port-site metastases; n: 19). The *p* value was calculated using Fisher’s exact test

Port-site metastases have been shown to increase morbidity in patients with ovarian cancer [10]. Interestingly in the PHC-LSC subgroup, patients with port-site metastases after LSC had a significant lower PFS (HR: 0.21; 95% CI 0.06–0.733, *p*: 0.014) and a trend towards a lower OS (HR: 0.298; 95% CI 0.073–1.216, *p*: 0.071), compared to patients with no port-site metastases after LSC (Fig. 3). There were 9 patients with port-site metastases in the non-tertiary-PHC-LSC subgroup and 1 patient in the tertiary-PHC-LSC group. Furthermore, a macroscopic complete resection was significantly more commonly achieved in the subgroup with LSC and no port site metastases compared with the subgroup with port-site metastases (*p*: 0.0052; Fig. 4).

The duration of the cytoreductive surgery between both groups differed (PHC-LSC group with port-site metastases median t: 180 min *vs*. PHC-LSC group without port-site metastases median time: 287 min; *p* < 0.05) with a significantly longer duration of surgery in the PHC-LSC group without port-site metastases.

## Discussion

Our study shows that patients with advanced ovarian cancer undergoing treatment solely at a tertiary center have clinical benefits, given the shorter time to CTx, the reduced rate of port-site metastases, which is reflected in an improved PFS for the latter. Our study further suggests that the time to CTx from first histological confirmation is an important independent prognostic factor.

The postoperative tumor burden after primary debulking surgery remains the most relevant prognostic factor for the outcome of patients with advanced ovarian cancer and macroscopic complete resection is more often achieved in tertiary specialized hospitals [11, 12]. Given that debulking surgery was solely performed at a single tertiary center in our study, the hospital type at which the PHC took place had expectedly no prognostic impact. Nonetheless, the non-tertiary PHC-LSC group had a longer time to cytoreductive surgery with more port-site metastases confirmed at the time of cytoreductive surgery.

Interestingly, a large retrospective study of 4097 patients showed that the time to CTx in patients with complete macroscopic resection after PDS (n: 1612) is an independent prognostic factor and a delay of more than 37 days results in worse OS compared to patients with a shorter time to CTx (HR for > 37 days: 1.43 (1.09–1.88) [13]. The authors conclude that adjuvant CTx should start within 5–6 weeks after debulking surgery. Most patients in our study (93%) received adjuvant CTx within six weeks and 83% within five weeks after cytoreductive surgery. Given that all patients received cytoreductive surgery at a specialized tertiary center with very similar access to adjuvant CTx, our cohort seems less suitable for assessing differences in time to CTx after debulking surgery.

However, comparing the time to adjuvant CTx between non-tertiary and tertiary PHC patients, counting from the day of the initial histological confirmation, there was a significant longer time to CTx for patients in the non-tertiary-PHC group. To the best of our knowledge, no study has yet investigated the potential prognostic relevance of a prolonged time to CTx beginning from the initial histological confirmation. We also confirmed the independent prognostic importance of the time to CTx in a multivariate analysis in the PHC group. Time to CTx has also been shown to be a surrogate marker for impaired prognosis in a large study of 45,000 patients with ovarian cancer because a delay in CTx of more than 35 days was associated with impaired survival [15].

Further studies may need to be performed to fully assess its clinical relevance. This has a urgent clinical implication as new clinical trials are investigating treating patients with advanced ovarian cancer before debulking surgery (“window of opportunity”) to maximize treatment effect and minimize time without treatment [14]. In our study, the median time to CTx (from initial histological confirmation) was 32 days in the tertiary-PHC group whereas it was 53 days in the non-tertiary-PHC group. It remains unclear whether this trend could have a prognostic impact in a larger cohort, particularly as there is no difference in the time to CTx from the date of surgical debulking in both subgroups. This further suggests that any delay in treatment results from the transfer between non-tertiary to the specialized tertiary hospital.

Several studies indicate a more favorable prognosis when diagnosis, surgery and treatment occur in tertiary specialized centers [16–19], emphasizing the importance that non-tertiary centers should not delay the referral of patients with a suspected ovarian cancer. Staging laparoscopy at non-tertiary centers may lack sufficient detail, hindering tertiary centers’ ability to assess if macroscopic complete resection is achievable through primary debulking surgery. Issues arise when descriptions of invasive tumor deposits (i.e. of the mesenteric root) are inadequate. Larger studies need to be conducted to adequately assess the factors that contribute to these differences. Nonetheless, our study points towards crucial clinical implications, given the shorter time to CTx, shorter time to surgical debulking and lower incidence of port-site metastases in the patients treated solely at the tertiary medical center.

Given their prognostic impact in our subgroup analysis, this is particularly true for the occurrence of port-site metastases, which are known morbidity driver in patients with ovarian cancer [10]. This was stated in a study with 214 patients with epithelial ovarian cancer who underwent diagnostic laparoscopy before debulking surgery and have had their port sites systematically resected during debulking surgery. They found that 100/214 patients (46,7%) had developed port-site metastases at the time of debulking surgery. The median time between the LSC and the debulking surgery was 25 days. They demonstrated that patients with port-site metastases had higher rates of wound healing disorders and overall increased morbidity.

Although the time to debulking surgery was similar in the PHC-LSC group with and without port-site metastases, we observed an improved outcome for the PHC-LSC patients without port metastases. However, this needs to be interpreted with caution as this was only seen in a sub analysis (n = 10) of our patient cohort. Interestingly, only one patient who underwent the PHC-LSC at the tertiary medical center developed a port-site metastases (with debulking surgery after 11 days), whereas nine patients developed port-site metastases who underwent PHC-LSC in a non-tertiary medical center (with debulking surgery after a median of 31 days). Although the median time to debulking surgery seems different, its impact remains controversial as a previous study did not confirm the time to surgical debulking to be an independent factor for the development of port-site metastases in ovarian cancer patients [20].

However, several studies suggest that the surgical expertise can have an important influence on the development of port-site metastases [21, 22]. This reflects our data as only one patient developed port-site metastases after LSC performed at the tertiary center but nine patients with port-site metastases from the non-tertiary medical centers [10, 23]. Although port-site metastases have shown to adversely impact outcome and morbidity, it might simply reflect advanced disease, aggressive tumor biology and potentially other yet unknown adverse factors. Hence, this should not discourage from performing diagnostic laparoscopic interventions for patients with suspect ovarian tumors, if clinically indicated. Nonetheless, this should be performed in tertiary centers if ovarian cancer is suspected so that the surgical evaluation is already performed by expert surgeons who can subsequently perform the debulking surgery [4, 24].

Despite the mono-center nature of our study, a potential regional bias and the medium-size patient cohort, our study has several strengths: the well-documented cases, long follow-up, high-quality treatment at a tertiary, cancer center and/or regional clinical partners. Furthermore, given the similar patients’ characteristics between the two major groups (PHC *vs.* PDS), one can conclude that differences in outcome may less likely be a result of different baseline clinical parameters. In addition, we performed a case–control matching in the PHC subgroup. In summary, this allows to robustly compare relevant clinical and prognostic parameters.

In conclusion, our study shows that patients with advanced ovarian cancer undergoing treatment solely at a tertiary center have some clinical benefits, given the shorter time to Ctx and reduced rate of port-site metastases, which is reflected in an improved PFS in this subgroup. Our study also contributes to the ongoing debate about the centralization of oncologic treatment in specialized, certified centers.

## Supplementary Information

Below is the link to the electronic supplementary material.

Supplementary file1 (TIFF 3533 kb)

Supplementary file2 (TIFF 2608 kb)

Supplementary file3 (TIFF 2265 kb)

## Data Availability

All relevant data and descriptions of statistical workflows are contained in the manuscript. Raw data can be made available from the first author (OliviaMariaApollonia.Starke@ukdd.de) upon reasonable request.

## List of abbreviations

OS: Overall survival
PFS: Progression-free survival
CI: Confidence interval
HR: Hazard ratio
CTx: Chemotherapy
FIGO: Fédération Internationale de Gynécologie et d’Obstétrique
PDS: Primary debulking surgery without prior diagnostic intervention for histologic confirmation
PHC: Diagnostic intervention for primary histological confirmation before debulking surgery
PHC-LSC: Primary histological confirmation via laparoscopy
LSC: Laparoscopy
HRD: Homologous recombination deficiency
BRCA: Breast cancer gene
