# Supplementary figures and images for: The prognostic influence of hospital type, method of first histological confirmation and time to chemotherapy in patients with advanced primary ovarian cancer
Source: Arch Gynecol Obstet. 2024 Dec 16;311(6):1627–35. doi: 10.1007/s00404-024-07832-4 (PMC12055883; doi:10.1007/s00404-024-07832-4)

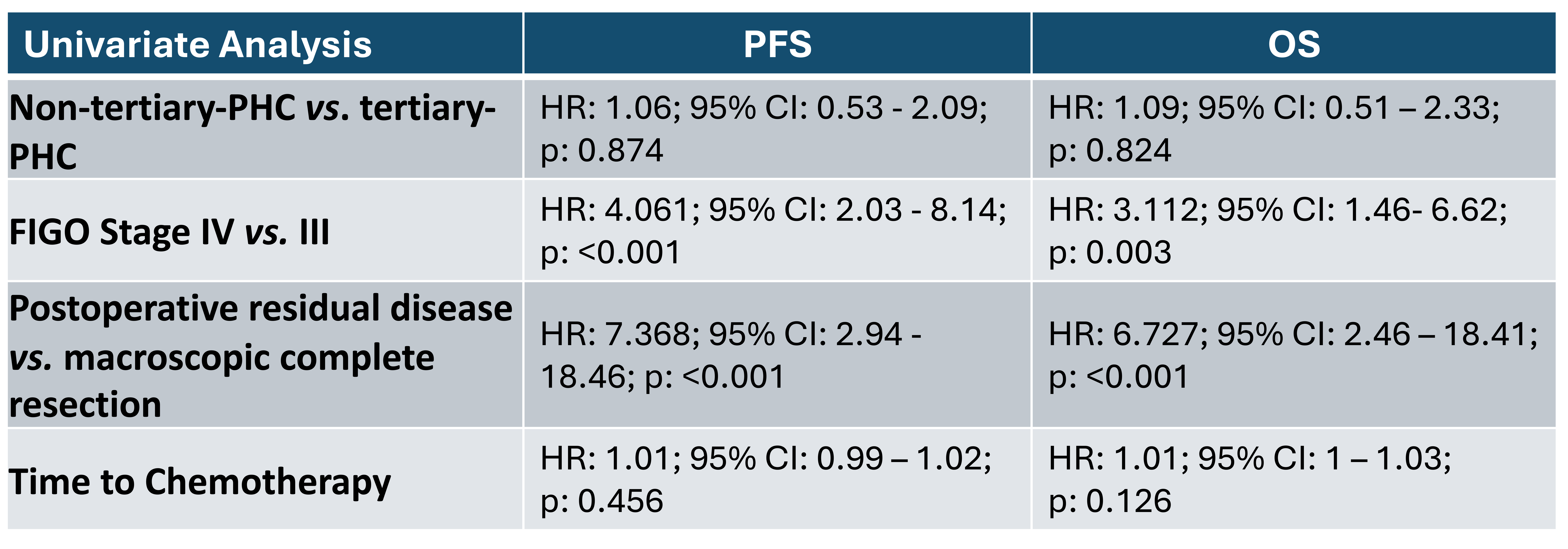

Supplement: Supplementary file 2 — Supplementary file2 (TIFF 2608 kb) [file 404_2024_7832_MOESM2_ESM.tiff]

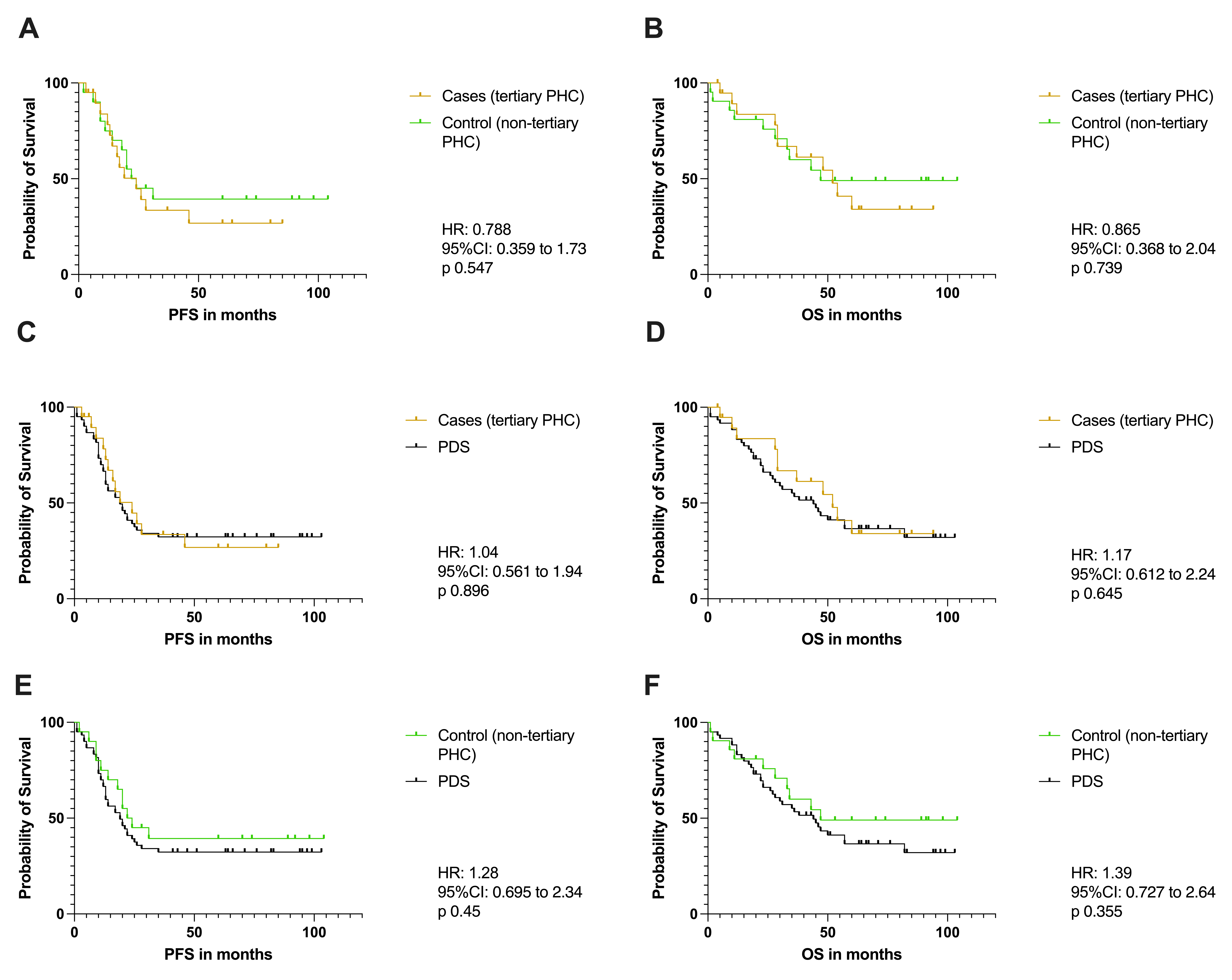

Supplement: Supplementary file 3 — Supplementary file3 (TIFF 2265 kb) [file 404_2024_7832_MOESM3_ESM.tiff]
